# Supplementary material for: A 6&1-FEH Encodes an Enzyme for Fructan Degradation and Interact with Invertase Inhibitor Protein in Maize (Zea mays L.)
Source: Int J Mol Sci. 2019 Aug 4;20(15):3807. doi: 10.3390/ijms20153807 (PMC6696269; doi:10.3390/ijms20153807)
Supplement: Supplementary file 1 [file ijms-20-03807-s001.pdf]

Supplementary Materials:

Table S1. Oligonucleotides used for PCR amplification and cloning. Included are primers for full length constructs (full); partial cDNAs for qPCR (qPCR); for heterologous expression of mature proteins for *P. pastoris* overexpression (pPICZ) and *E.coil* overexpression (pETG); for Gateway 2-step PCR (For pETG vector *E.coil* expression).

cDNA	Comment	Primer name	Primer sequence (5′-3′)
Zm-6&1-FEH1	Full gateway +/-Stop codon	FEH1GW-F	GGGGACAAGTTTGTACAAAAAAGCAGGCTCCATGAGGGCGCTTGGAACGGTT
		FEH1GW-R+	GGGGACCACTTTGTACAAGAAAGCTGGGTCTCAAGCGCCGTTCATGAGTGG
		FEH1GW-R-	GGGGACCACTTTGTACAAGAAAGCTGGGTCAGCGCCGTTCATGAGTGG
	qPCR	FEH1qPCR-F	TTGCGTGATATGGAATGGT
		FEH1qPCR-R	CCTAGTGGCATCTGAATC
	pPICZαA	FEH1pPICZ-F	AATCCGGAATTCTCTCATGTCGTCTACGAGAAC
		FEH1pPICZ-R	GACTAGTCTAGACCAGCGCCGTTCATGAGTGGCTT
Zm-INVINH1	Full gateway +/-Stop codon	Inh1GW-F	GGGGACAAGTTTGTACAAAAAAGCAGGCTCCATGAAGCTTCTGCAAGCTCTGT
		Inh1GW-R+	GGGGACCACTTTGTACAAGAAAGCTGGGTCTTACAACGCGGCCGTTACAGACA
		Inh1GW-R-	GGGGACCACTTTGTACAAGAAAGCTGGGTCCAACGCGGCCGTTACAGACAGC
Zm-ubiquitin	qPCR	UbiqPCR-F	CTCTTTCCCCAACCTCGTGTT
		UbiqPCR-R	ACGAGCGGCGTACCTTGA
Zm-actin	qPCR	ActinqPCR-F	CGATTGAGCATGGCATTGTCA
		ActinqPCR-R	CCCACTAGCGTACAACGAA
Zm-β-tubulin	qPCR	TubuqPCR-F	CCTATAACGCCACGCTCTCTGT
		TubuqPCR-R	CATTGTCCAGCACCATGCA
Gateway 2-step PCR pETG vector E.Coli expression		attB1_TEV_adaptor	GGGGACAAGTTTGTACAAAAAAGCAGGCTCTGAGAATCTTTATTTTCAGGGC
		attB2 adaptor	GGGGACCACTTTGTACAAGAAAGCTGGGT
